# Association of work-related and leisure-time physical activity with workplace food purchases, dietary quality, and health of hospital employees

**DOI:** 10.1186/s12889-019-7944-1

**Published:** 2019-11-27

**Authors:** Emily H. Feig, Douglas E. Levy, Jessica L. McCurley, Eric B. Rimm, Emma M. Anderson, Emily D. Gelsomin, Anne N. Thorndike

**Affiliations:** 1 Department of Psychiatry, Massachusetts General Hospital, Boston, MA USA; 2 Harvard Medical School, Boston, MA USA; 3 Mongan Institute Health Policy Research Center, Department of Medicine, Massachusetts General Hospital, Boston, MA USA; 4 Division of General Internal Medicine, Department of Medicine, Massachusetts General Hospital, 100 Cambridge Street, Boston, MA 02114 USA; 5 Department of Nutrition, Harvard T.H. Chan School of Public Health, Harvard University, Boston, MA USA; 6 Channing Division of Network Medicine, Department of Medicine, Brigham and Woman’s Hospital, Boston, MA USA; 7 Department of Nutrition and Food Services, Massachusetts General Hospital, Boston, MA USA

**Keywords:** Worksite wellness, Physical activity, Cardiometabolic risk, Work-related physical activity, Leisure-time physical activity, Sedentary time

## Abstract

**Background:**

While leisure-time physical activity (PA) has been associated with reduced risk of cardiometabolic disease, less is known about the relationship between work-related PA and health. Work-related PA is often not a chosen behavior and may be associated with lower socioeconomic status and less control over job-related activities. This study examined whether high work-related PA and leisure-time PA reported by hospital employees were associated with healthier dietary intake and reductions in cardiometabolic risk.

**Methods:**

This was a cross-sectional analysis of 602 hospital employees who used workplace cafeterias and completed the baseline visit for a health promotion study in 2016–2018. Participants completed the International Physical Activity Questionnaire and clinical measures of weight, blood pressure, HbA1c, and lipids. Healthy Eating Index (HEI) scores were calculated from two 24-h dietary recalls, and a Healthy Purchasing Score was calculated based on healthfulness of workplace food/beverage purchases. Regression analyses examined Healthy Purchasing Score, HEI, and obesity, hypertension, hyperlipidemia, and diabetes/prediabetes by quartile of work-related PA, leisure-time PA, and sedentary time.

**Results:**

Participants’ mean age was 43.6 years (SD = 12.2), 79.4% were female, and 81.1% were white. In total, 30.3% had obesity, 20.6% had hypertension, 26.6% had prediabetes/diabetes, and 32.1% had hyperlipidemia. Median leisure-time PA was 12.0 (IQR: 3.3, 28.0) and median work-related PA was 14.0 (IQR: 0.0, 51.1) MET-hours/week. Higher leisure-time PA was associated with higher workplace Healthy Purchasing Score and HEI (p’s < 0.01) and lower prevalence of obesity, diabetes/prediabetes, and hyperlipidemia (p’s < 0.05). Work-related PA was not associated with Healthy Purchasing Score, HEI, or cardiometabolic risk factors. Increased sedentary time was associated with lower HEI (*p* = 0.02) but was not associated with the workplace Healthy Purchasing Score.

**Conclusions:**

Employees with high work-related PA did not have associated reductions in cardiometabolic risk or have healthier dietary intake as did employees reporting high leisure-time PA. Workplace wellness programs should promote leisure-time PA and healthy food choices for all employees, but programs may need to be customized and made more accessible to meet the unique needs of employees who are physically active at work.

**Trial registration:**

This trial was prospectively registered with clinicaltrials.gov (Identifier: NCT02660086) on January 21, 2016. The first participant was enrolled on September 16, 2016.

## Introduction

Physical activity (PA) is predictive of many health benefits, including prevention of cardiovascular disease and diabetes [1]. Leisure-time PA is purposeful activity enacted often by individuals who also engage in other proactive healthy behaviors, including healthy dietary choices [2–5], and numerous studies have demonstrated the association of leisure-time PA with reduced risk for cardiometabolic disease [6–8]. Sedentary time has been shown to have powerful negative effects on health, independent of PA level [9–11], and has been associated with a less healthy diet, particularly in the context of television watching [2, 12–15]. Despite the strong associations of leisure-time PA and sedentary time with diet quality and health, less is known about how PA obtained as part of one’s job (work-related PA) impacts these outcomes.

Unlike leisure-time PA, work-related PA is often not a chosen behavior. Factors like lower socioeconomic status and less control over job-related activities are associated with job types that require more PA, and therefore work-related PA may be a marker for other negative health factors [16]. Most adults spend a large portion of their waking hours at work. If this time is spent engaging in healthy behaviors, such as eating a healthy diet, avoiding excessive sedentary behavior, and being physically active, one would expect a reduced disease risk. However, data have been mixed on the association of work-related PA with employees’ health. In some studies, high work-related PA has predicted better health, including lower rates of metabolic syndrome, lower obesity prevalence, and higher self-rated health [8, 17–19]. Other studies have shown that high work-related PA is associated with adverse health events, including coronary events and long term sickness absence [7, 20]. A recent meta-analysis of 193,696 participants from 17 studies found that men with high work-related PA had an 18% increased risk of all-cause mortality compared to those with low levels [21]. Although these analyses were inherently confounded by socioeconomic status, the authors argue that work-related PA may be one pathway for the higher mortality risks of those with lower socioeconomic status.

There are several explanations why work-related PA may not have the same health benefits as does leisure-time PA. Work-related and leisure-time PA have distinct characteristics. For example, work-related PA often requires heavy lifting and static and repetitive working postures. In contrast to leisure-time PA that is directly under one’s control and typically is performed in short bouts with breaks, workers must perform activity with low control over the tasks and speed and often are not given adequate recovery time [22]. Other lifestyle factors, such as diet and leisure-time PA, may play a more important role in health than work-related PA.

The present study examined how domains of PA (i.e., work-related, leisure-time) and sedentary time related to healthfulness of objectively measured cafeteria purchases, overall dietary quality, and cardiometabolic health measures of 602 hospital employees enrolled in a randomized controlled trial (*ChooseWell 365)* to promote healthy lifestyle [23]. Hospital employees perform jobs that require varying levels of PA, ranging from sedentary desk jobs to maintenance and security jobs requiring lifting and long bouts of walking. Therefore a hospital workplace is ideal for exploring how work-related and leisure-time PA and sedentary time are related to employees’ lifestyle choices and health. We hypothesized that [1] employees with higher leisure-time PA would have healthier cafeteria purchases and overall dietary quality and have lower cardiometabolic risk factors [2]; employees with higher work-related PA would not have healthier dietary quality but would have lower cardiometabolic risk factors due to the direct benefits of being more active; and [3] independent of total PA, employees with more sedentary time would have lower diet quality and higher cardiometabolic risk factors.

## Methods

### Participants and setting

This study included 602 employees of Massachusetts General Hospital (MGH) in Boston, Massachusetts who completed a baseline visit as part of a randomized controlled trial testing a worksite dietary intervention (*ChooseWell 365*) between September 2016 and February 2018 [23]. The study tracked participants’ workplace cafeteria purchases before, during, and after the intervention. To be included in the study, employees had to be 20–75 years old and make cafeteria purchases at least 4 times per week for at least 6 weeks during a 12-week period prior to study recruitment. Employees were ineligible if they were pregnant, wanted to gain weight, were currently participating in a weight loss study, had weight loss surgery in the past 6 months, had an eating disorder history, were employed in the cafeteria, or had plans to terminate their employment at the hospital in the next year. Employees may have worked on either weekdays or weekends, but there was no available information about which days of the week individual employees worked. The present study was a cross-sectional analysis of baseline data from the trial. Participants completed two 24-h dietary recalls online and attended a clinical visit. All data used in this study were collected prior to randomization and any intervention procedures.

### Measures

#### Clinical assessment

Participants attended a clinic visit at which their height, weight, and blood pressure were measured, and blood was drawn for a fasting lipid panel, glucose, and hemoglobin A1c tests. Body mass index (BMI) was used to categorize weight status (obese = BMI ≥ 30 kg/m^2^, not obese = BMI < 30 kg/m^2^). Hypertension was defined as at least one of the following: (a) self-reported hypertension or high blood pressure diagnosis by a medical professional; (b) self-reported use of prescription antihypertensive medication; (c) study measurement of systolic blood pressure ≥ 150 mmHg; and/or (d) diastolic blood pressure ≥ 90 mmHg [24]. We chose a systolic blood pressure cut-off that was higher than the guidelines (> 130 mmHg) to be conservative in our definition of hypertension for participants who had not previously been diagnosed with high blood pressure. Prediabetes/diabetes was defined as at least one of the following: (a) self-reported diabetes or prediabetes diagnosis by a medical professional; (b) self-reported use of prescription medication for diabetes; and/or (c) study measurement of HbA1c ≥ 5.7 [25]. Type 1 and 2 diabetes were not differentiated. Hyperlipidemia was defined as at least one of the following: (a) self-reported diagnosis of high cholesterol/hyperlipidemia; (b) self-reported use of prescription medication for high cholesterol; and/or (c) study measurement of fasting total cholesterol ≥220; low density lipoprotein ≥160; or triglycerides ≥180 [26].

#### Administrative data

Participant job type was collected from the hospital’s human resources department. Job titles were combined into four categories determined based on educational attainment needed for the type of work: [1] service workers (manual and/or unskilled laborers)/administrative assistants [2]; craft/technicians (e.g., radiology technicians) [3]; management/professionals (e.g., social workers, nurses, hospital managers); and [4] MDs/PhDs (e.g., physicians, researchers).

#### Dietary intake

Dietary quality was assessed using Automated Self-Administered 24-h (ASA24) dietary recall surveys. This tool was developed by the National Cancer Institute and uses multi-level probes to guide respondents through reporting their intake over the prior 24 h [27]. In most cases (93.7%), two ASA24 recalls were collected on non-consecutive days and a Healthy Eating Index (HEI) score was calculated using their average [28].. In the small number of cases where only one ASA24 was completed (6.3%), the HEI was based on that recall. The HEI score measures an individual’s compliance with dietary recommendations from the United States Department of Agriculture (USDA) Guidelines for America. The most recent HEI (HEI-2015) was used in this study. Scores range from 0 to 100, with higher scores signifying better compliance with dietary guidelines. Americans had an average HEI of 59 out of 100 in 2013–2014, based on data from the National Health and Nutrition Examination Study [29].

#### Worksite cafeteria purchases

All MGH hospital cafeterias use traffic light food labeling to provide information about the healthfulness of food and drink items (green = healthy, yellow = less healthy, red = unhealthy). The labeling algorithm was developed by hospital nutrition staff and was based on USDA dietary guidelines [30], using positive and negative nutritional criteria to rate all food/beverage items. Positive criteria included having the main ingredient be: [1] a fruit or vegetable, [2] a whole grain, and/or [3] a lean protein, plant-based meat substitute, or low-fat dairy. Negative criteria included: [1] saturated fat content of ≥5 g per entrée or ≥ 2 g per non-entrée item, condiment, or beverage, and/or [2] caloric content ≥500 kcal per entrée, ≥ 200 kcal per non-entrée food item, or ≥ 100 kcal per condiment or beverage. Items were categorized as green if they had more positive than negative criteria; those with equal positive and negative criteria, with only one negative criterion, or with no positive or negative criteria were labeled yellow; and those with multiple negative criteria and no positive criteria were labeled red. While all items available in the salad bar were rated individually, color labels were assigned to each salad *purchase* based on weight for study participants (green: salad < 16 oz; yellow: salad ≥16 oz) [23]. Each cafeteria has permanent, highly visible signage explaining the labeling system.

Cafeteria purchases and the associated traffic light label colors were retrospectively collected from the cafeteria cash register data system for the 3 months prior to each participant’s study enrollment date. A Healthy Purchasing Score was calculated based on the weighted proportion of items purchased that were labeled red, yellow, or green to reflect overall healthfulness of a participant’s purchases over those 3 months [31]. The proportion of red items was multiplied by 0, the proportion of yellow items was multiplied by 0.5, and the proportion of green items was multiplied by 1. The sum of these values was the Healthy Purchasing Score, ranging from 0 (100% red items, least healthy) to 1 (100% green items, healthiest). For example, if an employee’s 3-month baseline purchases were 20% red, 50% yellow, and 30% green-labeled items, the Healthy Purchasing Score would be: (0.2 red × 0) + (0.5 yellow × 0.5) + (0.3 green × 1) = 0.55.

#### International physical activity questionnaire (IPAQ) long version

This validated and commonly used measure asks about one’s last 7 days of physical activity, split by domain in which the activity occurred: job-related, transportation, housework/house maintenance/caring for family, and recreation/sport/leisure [32]. Sedentary time is assessed as time spent sitting across all domains. The present study included job-related PA (“work-related PA”), recreation/sport/leisure PA (“leisure-time PA”), sedentary time, and total PA (summed across all domains). We followed the scoring guidelines that recommend truncating time spent on each level of physical activity (walking, moderate, and vigorous) at 180 min per day to avoid reporting errors [33]. Totals were calculated in terms of metabolic equivalents of hours per week (MET-hours), by assigning a multiplier to activity based on its intensity (walking = 3.3, moderate activity = 4, vigorous activity = 8). The IPAQ has acceptable measurement properties and high reliability (α = 0.80) [32].

### Statistical analyses

Analyses were conducted with Stata version 15.1 (Stata Corporation, College Station, TX). Medians were calculated to summarize work-related and leisure-time PA overall and by quartile. Multivariate regression models tested whether leisure-time PA, work-related PA, and sedentary time were associated with each other, and whether they each were associated with Healthy Purchasing Score and HEI, adjusting for age, sex, race, ethnicity, education, season at time of assessment, and PA (leisure-time analyses adjusted for work-related PA, work-related analyses adjusted for leisure-time PA, sedentary time analyses adjusted for total PA). Leisure-time PA, work-related PA, and sedentary time, when used as covariates, were divided into quartiles and entered into the models using the medians of each quartile to flexibly and efficiently model their non-normal distributions. Logistic regression analyses tested prevalence of obesity, prediabetes/diabetes, hypertension, and hyperlipidemia, adjusting for the same variables as above in addition to the HEI, by quartile of each type of PA. Regression-adjusted mean outcomes were calculated using Stata’s “predict” command as the mean of predicted probabilities evaluated assuming all subjects had the median value of a particular quartile while retaining their observed characteristics on other covariates. Participants with missing data were excluded from analyses that included the missing variable.

## Results

Characteristics of the full sample of participants, and by quartile of work-related PA and leisure-time PA, are presented in Tables 1 and 2 respectively. Sample sizes in each quartile differed somewhat, which occurs when multiple participants have tied values near the edge of a quartile category. Participants were 43.6 years old on average (SD = 12.2), and the majority were female, white, and had at least a college degree. BMI was 28.3 kg/m^2^ (SD = 6.5) on average, and 30.2% had obesity. The prevalence of hypertension, prediabetes/diabetes, and hyperlipidemia were 20.6, 26.6, and 32.1%, respectively. Demographic information related to ethnicity and/or education was missing for 14 participants (2.3%), and HEI was missing for 26 participants (4.3%). Participants with missing data were excluded from analyses that included that variable, such that analyses including the HEI had an N of 562 and those without the HEI had an N of 588.

**Table 1:** Characteristics of participants overall and by quartile of work-related physical activity

		Work-related physical activity quartiles			
Variable	Total *N* = 602	Q1 (lowest) *N* = 174 0 met-hours per week	Q2 *N* = 127 1–14 met-hours per week	Q3 *N* = 151 14–51 met-hours per week	Q4 (highest) *N* = 150 52–254 met-hours per week
	**M (SD)**				
Age (years)	43.6 (12.2)	45.2 (12.0)	43.9 (12.5)	43.5 (12.2)	41.7 (12.2)
	**N (%)**				
Gender					
Male	124 (20.6)	29 (16.7)	17 (13.4)	37 (24.5)	41 (27.3)
Female	478 (79.4)	145 (83.3)	110 (86.6)	114 (75.5)	109 (72.7)
Race					
White	488 (81.1)	137 (78.7)	101 (79.5)	127 (84.1)	123 (82.0)
Black	54 (9.0)	16 (9.2)	15 (11.8)	12 (8.0)	11 (7.3)
Asian	27 (4.5)	10 (5.8)	8 (6.3)	4 (2.7)	5 (3.3)
Other/Not reported	33 (5.5)	11 (6.3)	3 (2.4)	8 (5.3)	11 (7.3)
Ethnicity					
Non-Hispanic/Latino/a	556 (92.4)	162 (93.1)	116 (91.3)	142 (94.0)	136 (90.7)
Hispanic/Latino/a	34 (5.6)	9 (5.2)	8 (6.3)	7 (4.6)	10 (6.7)
Not reported	12 (2.0)	3 (1.7)	3 (2.4)	2 (1.3)	4 (2.7)
Job Type					
Administrative/Service	84 (14.0)	34 (19.5)	19 (15.0)	12 (8.0)	19 (12.7)
Craft/Technicians	67 (11.1)	16 (9.2)	8 (6.3)	13 (8.6)	30 (20.0)
Management/ Professionals	377 (62.6)	100 (57.5)	74 (58.3)	106 (70.2)	97 (64.7)
MDs/PhDs	74 (12.3)	24 (13.8)	26 (20.5)	20 (13.3)	4 (2.7)
Education Level					
High School/Some College	75 (12.5)	30 (17.2)	10 (7.9)	14 (9.3)	21 (14.0)
College Degree	240 (39.9)	56 (32.2)	44 (34.6)	60 (39.7)	80 (53.3)
Graduate Degree	284 (47.2)	88 (50.6)	73 (57.5)	76 (50.3)	47 (31.3)
Not reported	3 (0.5)	0 (0.0)	0 (0.0)	1 (0.7)	2 (1.3)
Current Smoker	17 (2.8)	3 (1.7)	2 (1.6)	6 (4.0)	6 (4.0)

**Table 2:** Characteristics of participants by quartile of leisure-time physical activity

	Leisure-time physical activity quartiles			
Variable	Q1 (lowest) *N* = 167 0–3 met-hours per week	Q2 *N* = 134 4–12 met-hours per week	Q3 *N* = 151 12–28 met-hours per week	Q4 (highest) *N* = 150 28–214 met-hours per week
	**M (SD)**			
Age	44.9 (12.0)	44.0 (12.0)	42.3 (12.2)	43.2 (12.6)
	**N (%)**			
Gender				
Male	31 (18.6)	28 (20.9)	30 (19.9)	35 (23.3)
Female	136 (87.4)	106 (89.1)	121 (80.1)	115 (76.7)
Race				
White	127 (76.1)	111 (82.2)	127 (84.7)	123 (82.0)
Black	22 (13.2)	11 (8.2)	10 (6.7)	11 (7.3)
Asian	7 (4.2)	6 (4.4)	7 (4.7)	7 (4.7)
Other/Not reported	11 (6.6)	7 (5.2)	6 (4.0)	9 (6.0)
Ethnicity				
Non-Hispanic/Latino/a	151 (90.4)	128 (95.5)	140 (92.7)	137 (91.3)
Hispanic/Latino/a	11 (6.6)	5 (3.7)	10 (6.6)	8 (5.3)
Not reported	5 (3.0)	1 (0.7)	1 (0.7)	5 (3.3)
Job Type				
Administrative/Service	32 (19.2)	18 (13.3)	21 (14.0)	13 (8.7)
Craft/Technicians	24 (14.4)	15 (11.2)	12 (8.0)	16 (10.7)
Management/ Professionals	92 (55.1)	76 (56.7)	99 (66.0)	110 (73.3)
MDs/PhDs	19 (11.4)	26 (19.4)	18 (12.0)	11 (7.3)
Education Level				
High School/Some College	26 (15.6)	16 (11.9)	18 (11.9)	15 (10.0)
College Degree	76 (45.5)	54 (40.3)	51 (33.8)	59 (39.3)
Graduate Degree	64 (38.3)	65 (48.5)	79 (52.3)	76 (50.7)
Not reported	1 (0.6)	0 (0.0)	2 (1.3)	0 (0.0)
Current Smoker	7 (4.2)	6 (4.5)	2 (1.3)	2 (1.3)

As shown in Table 3, participants reported a higher median work-related than leisure-time PA, which was driven largely by higher MET-hours reported by those in the top quartile of PA. The intensity levels of work-related and leisure-time PA also differed. The largest proportion of leisure-time METs came from vigorous activity (47.7%), followed by walking (40.4%) and moderate activity (12.0%; data not shown). The largest proportion of work-related activity was from walking (60.8%), followed by moderate activity (23.1%) and then vigorous activity (16.1%). Work-related and leisure-time PA were positively associated, controlling for covariates (b = 0.44; 95% CI: 0.27, 0.61; *p* < 0.001) such that each additional hour of leisure-time PA was associated with 0.44 more hours of work-related PA per week (data not shown). Additionally, sedentary time was negatively associated with work-related PA (b = − 7.61; 95% CI; − 9.16, − 6.05; *p* < 0.001) and leisure-time PA (− 1.20; − 2.01, − 0.39; *p* = 0.004), controlling for covariates, such that each additional hour of sedentary time per day was associated with 7.61 fewer hours/week of work-related PA and 1.20 fewer hours/week of leisure-time PA.

**Table 3:** Median and interquartile range of work-related PA, leisure-time PA, and sedentary time by quartile

	Physical activity quartiles				
Variable	Total	Q1 (lowest)	Q2	Q3	Q4 (highest)
	Median (IQR)				
Work METs	14.0 (0.0, 51.1)	0.0 (0.0)	5.5 (2.8, 9.7)	29.7 (18.5, 41.7)	93.0 (67.0, 137.7)
Leisure METs	12.0 (3.3, 28.0)	0.0 (0.0, 99.0)	8.0 (6.6, 10.6)	19.9 (16.0, 24.0)	43.5 (35.1, 59.1)
Sedentary time	5.3 (3.7, 7.1)	2.7 (2.0, 3.4)	4.6 (4.1, 4.9)	6.1 (5.7, 6.6)	8.5 (8.0, 10.0)

Work-related and leisure-time PA measured as MET-hours per week. Sedentary time measured as hours per day

**Fig. 1:**
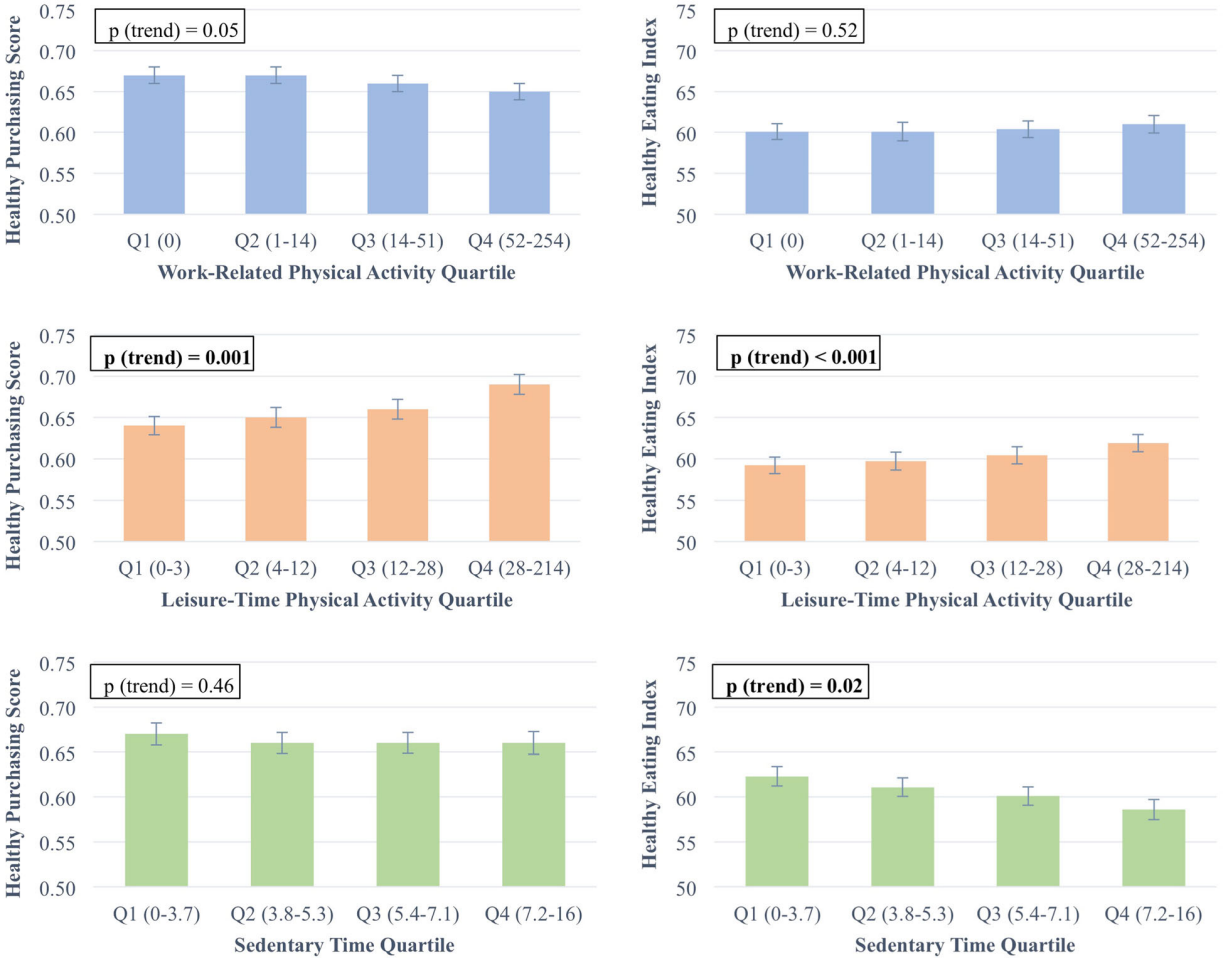
Associations between dietary health measures and physical activity measures. All analyses adjusted for age, sex, race, ethnicity, education, and season. Error bars reflect standard error. *P*-values refer to trend across quartiles; bold indicates a statistically significant difference between quartiles. **a** Healthy Purchasing Score by work-related PA quartiles, also adjusted for leisure-time PA. Numbers in parentheses reflect range of work-related MET-hours per week in each quartile. *N* = 588. **b** HEI by work-related PA quartiles, also adjusted for leisure-time PA. Numbers in parentheses reflect range of work-related MET-hours per week in each quartile. *N* = 562. **c** Healthy Purchasing Score by leisure-time PA quartiles, also adjusted for work-related PA. Numbers in parentheses reflect range of leisure-time MET-hours per week in each quartile. *N* = 588. **d** HEI by leisure-time PA quartiles, also adjusted for leisure-time PA. Numbers in parentheses reflect range of leisure-time MET-hours per week in each quartile. *N* = 562. **e** Healthy Purchasing Score by sedentary time quartiles, also adjusted for total PA. Numbers in parentheses reflect range of sedentary hours per day in each quartile. *N* = 588. **f** HEI by sedentary time quartiles, also adjusted for total PA. Numbers in parentheses reflect range of sedentary hours per day in each quartile. *N* = 562.

The associations between the categories of PA and healthy eating are illustrated in Fig. 1. Work-related PA was not associated with Healthy Purchasing Score or HEI. Leisure-time PA was positively associated with both the Healthy Purchasing Score and the HEI; regression-adjusted means for lowest versus highest quartiles were 0.64 and 0.69 for the HPS (*p* < 0.001) and 59.2 and 61.9 for HEI (*p* < 0.001). Increased sedentary time was associated with lower HEI (lowest quartile 62.3 and highest quartile 58.6, *p* = 0.02) but was not associated with Healthy Purchasing Score.

Work-related PA was not significantly associated with cardiometabolic risk factors, although there was a nonsignificant trend toward higher rates of hypertension, prediabetes/diabetes, and obesity with higher work-related PA (Table 4). Leisure-time PA was negatively associated with prevalence of prediabetes/diabetes (*p* = 0.001), hyperlipidemia (*p* = 0.016), and obesity (*p* < 0.001), controlling for covariates. Sedentary time was not associated with risk factors.

**Table 4:** Adjusted rates of cardiometabolic conditions by PA quartile

	Q1 (lowest)	Q2	Q3	Q4 (highest)	
Work-related PA	**%**	**%**	**%**	**%**	**p**
Hypertension	18.0	18.3	19.9	24.3	0.06
Prediabetes/Diabetes	24.3	24.5	25.7	29.0	0.06
Hyperlipidemia	33.7	33.4	31.8	27.9	0.10
Obesity	27.6	27.9	29.2	32.8	0.27
Leisure-time PA	**%**	**%**	**%**	**%**	**p**
Hypertension	21.0	20.8	20.5	19.8	0.19
Prediabetes/Diabetes	30.4	28.4	25.7	20.8	**0.001**
Hyperlipidemia	33.8	32.8	31.4	28.6	**0.016**
Obesity	36.6	33.3	28.7	20.1	**< 0.001**
Sedentary time	**%**	**%**	**%**	**%**	**p**
Hypertension	21.2	20.7	20.3	19.7	0.99
Prediabetes/Diabetes	22.0	24.5	26.7	30.2	0.14
Hyperlipidemia	30.4	31.1	31.7	32.5	0.95
Obesity	25.7	28.1	30.2	33.5	0.16

Adjusted for age, sex, race, ethnicity, education, season, and Healthy Eating Index. Work-related analyses controlled for leisure-time PA and leisure-time analyses controlled for work-related PA. *P***-**value refers to trend across quartiles; bold indicates a statistically significant difference between quartiles. *N* = 562

## Discussion

We found that higher work-related PA was not associated with healthier worksite purchases, better overall dietary quality, or reduced cardiometabolic risk factors. As expected, higher leisure-time PA was strongly associated with healthier purchases at work, healthier overall dietary quality, and lower risk of obesity, prediabetes/diabetes, and hyperlipidemia. Employees who reported high work-related PA tended to report higher MET-hours than did those who reported high leisure-time PA. These findings suggest that the types of PA obtained from job-related activities may not provide the health benefit of the types of PA done during leisure time. High work-related PA may also be an indicator of other unhealthy behaviors that warrant targeted intervention.

The results of this study are consistent with prior studies of PA domains and health measures showing that leisure-time PA is more strongly associated with better health than is work-related PA. Associations between domain-specific PA and diet have been less extensively studied. While a substantial body of work has found that individuals with more leisure-time PA have a healthier diet [2–5], no prior studies to our knowledge have used an objective measure such as cafeteria purchases to examine how dietary choices relate to PA. While the relationship in the present study did not reach statistical significance, there was a trend toward less healthy cafeteria purchases for those with higher work-related PA (*p* = 0.050). This may explain, in part, why employees with greater work-related PA do not have lower cardiometabolic risk factors. The physical benefits achieved by being active on the job may need to be paired with a healthier diet to lead to measurable change in these risk factors, and it could be that a tendency toward a less healthy diet is offsetting benefits from increased PA.

We found that employees who reported greater sedentary time had worse overall dietary quality but little difference in the healthfulness of workplace food choices. These findings reflect prior studies that found television watching as a mechanism by which sitting and poor diet are often linked [2, 12–14]. It may be that individuals’ sitting time at home is related to unhealthy diet more so than sitting at work. When studies assessed sedentary time for television watching and work sitting separately, television watching was consistently associated with higher biomarkers for cardiovascular disease/diabetes whereas sitting at work had few significant associations with negative health markers, and only for men [12]. Although the present study assessed sedentary time overall rather than by work or home domain, findings suggest that diet associated with sedentary behavior at home, rather than at work, may help to explain the discrepant associations with domain-specific sedentary time and health outcomes. Further work that discriminates sedentary time by setting is needed to confirm this hypothesis.

There was a lack of association between work-related PA and health outcomes, with a nonsignificant trend toward higher cardiometabolic risk factors in employees with higher work-related PA. This may be related to differences in the types of exercise done at work compared to during leisure time [22]. For example, as outlined by Holtermann and colleagues, work-related PA that includes heavy lifting and remaining in a static posture could elevate blood pressure. These effects are worsened when the activity is performed without sufficient recovery time and without worker control over the working environment, schedule, or tasks. Further, work-related PA tends to be of too low intensity and too long duration [22]. Additionally, heavy lifting at work has been associated with increased risk of myocardial infarction, whereas aerobic activity at work has been associated with decreased risk [34]. These differences may explain why, even though employees reported more work-related PA than leisure-time PA overall, beneficial associations with cardiometabolic risk factors were not seen with work-related PA. In our study we found that 48% of leisure-time METs were earned from vigorous activity, and only 17% of work-related METs were at the vigorous level. It is known that moderate-to-vigorous activity procures strong health benefits [35, 36], whereas the benefits from light activity are less established. Light activity may be more beneficial for older adults, whereas for younger individuals at least a moderate intensity is needed to protect against negative health outcomes [37]. In a relatively young employee population, light activity done throughout the workday is likely insufficient to produce major health benefits. Although we controlled for socioeconomic status using the available education and job type variables, it could also be that unmeasured social and other risk factors explain the trend seen toward higher cardiometabolic risk in employees with high work-related PA.

Strengths of this study include the ability to examine the relationships of different PA types with objective measurement of workplace food purchases and biometric health markers. In addition, the sample includes a large and relatively diverse sample of employees across a range of job types and requirements. However, this study also has limitations. All data are cross-sectional, thus causality cannot be determined from these results. It is unknown whether participants ate all the food they purchased at a cafeteria, or what other foods they may have eaten at work that were not purchased at a cafeteria, although the Healthy Purchasing Score was significantly associated with HEI in a previous study [31]. Self-reported PA with measures such as the IPAQ tends to be overestimated compared to objective measures. In particular, occupational activities can be challenging to assess with the IPAQ due to the common intermittent nature of such activities, spread over long periods of time. However, validation studies suggest that the IPAQ is a moderately good measure of occupational physical activity [38]. Finally, the sample included employees at one urban hospital that may not be representative of employees in non-hospital workplaces.

## Conclusions

In conclusion, higher work-related PA was not associated with healthier food purchases at work or overall diet quality and, in contrast to our hypothesis, was not associated with better cardiometabolic health. Consistent with prior research, employees’ leisure-time PA was strongly related to healthier dietary intake and lower cardiometabolic risk factors. Although employees with high work-related PA had higher overall PA compared to employees with high leisure time PA, results of this study suggest that PA earned on the job may not alone be sufficient for procuring health benefits. Other factors often seen in those with high work-related PA (e.g., lower socioeconomic status, less control over PA, less healthy diet) likely exert a stronger effect on health outcomes than does work-related PA itself. Further, by collapsing activity across domains into one single metric, important aspects of PA as it relates to health may be lost. It is important that workplace wellness interventions promote leisure-time PA and healthy food choices of employees with all levels of work-related PA. Such programs may need to be customized and made more accessible to meet the unique needs of employees with high work-related PA.

## Data Availability

The datasets analyzed during the current study are not publicly available because the study is ongoing but will be available from the corresponding author upon reasonable request after the final study follow-up is completed.

## Abbreviations

ASA24: Automated Self-Administered 24-h recall
BMI: Body mass index
HEI: Healthy Eating Index
IPAQ: International Physical Activity Questionnaire
IQR: Interquartile range
MET: Metabolic equivalent
MGH: Massachusetts General Hospital
PA: Physical activity

## References

[1] World Health Organization. Global health risks: Mortality and burden of disease attributable to selected major risks. Geneva: WHO Press; 2009.

[2] Charreire H, Kesse-Guyot E, Bertrais S, Simon C, Chaix B, Weber C (2011). Associations between dietary patterns, physical activity (leisure-time and occupational) and television viewing in middle-aged French adults. Br J Nutr.

[3] Simoes EJ, Byers T, Coates RJ, Serdula MK, Mokdad AH, Heath GW (1995). The association between leisure-time physical activity and dietary fat in American adults. Am J Public Health.

[4] Monfort-Pires M, Salvador EP, Folchetti LD, Siqueira-Catania A, Barros CR, Ferreira SRG (2014). Diet quality is associated with leisure-time physical activity in individuals at cardiometabolic risk. J Am Coll Nutr.

[5] Oppert J-M, Thomas F, Charles M-A, Benetos A, Basdevant A, Simon C (2008). Leisure-time and occupational physical activity in relation to cardiovascular risk factors and eating habits in French adults. Public Health Nutr.

[6] Huai P, Han H, Reilly KH, Guo X, Zhang J, Xu A (2016). Leisure-time physical activity and risk of type 2 diabetes: a meta-analysis of prospective cohort studies. Endocrine.

[7] Clays E, De Bacquer D, Janssens H, De Clercq B, Casini A, Braeckman L (2013). The association between leisure time physical activity and coronary heart disease among men with different physical work demands: a prospective cohort study. Eur J Epidemiol.

[8] Abu-Omar K, Rütten A (2008). Relation of leisure time, occupational, domestic, and commuting physical activity to health indicators in Europe. Prev Med (Baltim).

[9] Seguin R, Buchner DM, Liu J, Allison M, Manini T, Wang CY (2014). Sedentary behavior and mortality in older women: the women’s health initiative. Am J Prev Med.

[10] Matthews CE, George SM, Moore SC, Bowles HR, Blair A, Park Y (2012). Amount of time spent in sedentary behaviors and cause-specific mortality in US adults. Am J Clin Nutr.

[11] De Rezende LFM, Lopes MR, Rey-Loṕez JP, Matsudo VKR, Luiz ODC (2014). Sedentary behavior and health outcomes: an overview of systematic reviews. PLoS One.

[12] Pereira SM, Ki M, Power C (2012). Sedentary behaviour and biomarkers for cardiovascular disease and diabetes in mid-life: the role of television-viewing and sitting at work. PLoS One.

[13] Wagner A, Dallongeville J, Haas B, Ruidavets JB, Amouyel P, Ferrières J (2012). Sedentary behaviour, physical activity and dietary patterns are independently associated with the metabolic syndrome. Diabetes Metab.

[14] Utter J, Neumark-Sztainer D, Jeffery R, Story M (2003). Couch potatoes or French fries: are sedentary behaviors associated with body mass index, physical activity, and dietary behaviors among adolescents?. J Am Diet Assoc.

[15] Gillman MW, Pinto BM, Tennstedt S, Glanz K, Marcus B, Friedman RH (2001). Relationships of physical activity with dietary behaviors among adults. Prev Med (Baltim).

[16] Beenackers MA, Kamphuis CBM, Giskes K, Brug J, Kunst AE, Burdorf A (2012). Socioeconomic inequalities in occupational, leisure-time, and transport related physical activity among European adults: A systematic review. Int J Behav Nutr Phys Act.

[17] Chu AHY, Moy FM (2013). Associations of occupational, transportation, household and leisure-time physical activity patterns with metabolic risk factors among middle-aged adults in a middle-income country. Prev Med (Baltim).

[18] Chau JY, van der Ploeg HP, Merom D, Chey T, Bauman AE (2012). Cross-sectional associations between occupational and leisure-time sitting, physical activity and obesity in working adults. Prev Med (Baltim).

[19] Hu G, Jousilahti P, Borodulin K, Barengo NC, Lakka TA, Nissinen A (2007). Occupational, commuting and leisure-time physical activity in relation to coronary heart disease among middle-aged Finnish men and women. Atherosclerosis.

[20] Holtermann A, Hansen JV, Burr H, Søgaard K, Sjøgaard G (2012). The health paradox of occupational and leisure-time physical activity. Br J Sports Med.

[21] Coenen P, Huysmans MA, Holtermann A, Krause N, Van Mechelen W, Straker LM (2018). Do highly physically active workers die early? A systematic review with meta-analysis of data from 193 696 participants. Br J Sports Med.

[22] Holtermann A, Krause N, Van Der Beek AJ, Straker L (2018). The physical activity paradox: six reasons why occupational physical activity (OPA) does not confer the cardiovascular health benefits that leisure time physical activity does. Br J Sports Med.

[23] Levy DE, Gelsomin ED, Rimm EB, Pachucki M, Sanford J, Anderson E (2018). Design of ChooseWell 365: randomized controlled trial of an automated, personalized worksite intervention to promote healthy food choices and prevent weight gain. Contemp Clin Trials.

[24] Whelton P, Carey R, Aronow W, Casey D, Collins K, Dennison Himmelfarb C (2018). 2017 ACC/AHA/AAPA/ABC/ACPM/AGS/APhA/ASH/ASPC/NMA/PCNA guideline for the prevention, detection, evaluation, and Management of High Blood Pressure in adults: a report of the American College of Cardiology/American Heart Association task force on clinical Pr. Hypertension.

[25] American Diabetes Association (2013). Standards of medical care in diabetes - 2013. Diabetes Care.

[26] Grundy S, Stone N, Bailey A, Beam C, Birtcher K, Blumenthal R (2019). AHA/ACC/AACVPR/AAPA/ABC/ACPM/ ADA/AGS/APhA/ASPC/NLA/PCNA guideline on the management of blood cholesterol: a report of the American College of Cardiology/American Heart Association task force on clinical practice guidelines. J Am Coll Cardiol.

[27] Subar AF, Kirkpatrick SI, Mittl B, Zimmerman TP, Thompson FE, Bingley C (2012). The automated self-administered 24-hour dietary recall (ASA24): a resource for researchers, clinicians, and educators from the National Cancer Institute. J Acad Nutr Diet.

[28] Krebs-Smith SM, Pannucci TRE, Subar AF, Kirkpatrick SI, Lerman JL, Tooze JA (2018). Update of the healthy eating index: HEI-2015. J Acad Nutr Diet.

[29] U.S. National Center for Health Statistics. What We Eat in America/National Health and Nutrition Examination Survey, 2013–2014: Healthy Eating Index-2015. [Internet]. 2018. Available from: https://www.cnpp.usda.gov/healthyeatingindex

[30] Thorndike AN, Sonnenberg L, Riis J, Barraclough S, Levy DE (2012). A 2-phase labeling and choice architecture intervention to improve healthy food and beverage choices. Am J Public Health.

[31] McCurley J, Levy D, Rimm E, Gelsomin E, Anderson E, Sanford J (2019). Association of worksite food purchases and employees’ overall dietary quality and health. Am J Prev Med.

[32] Craig CL, Marshall AL, Sjöström M, Bauman AE, Booth ML, Ainsworth BE (2003). International physical activity questionnaire: 12-country reliability and validity. Med Sci Sports Exerc.

[33] IPAQ. Guidelines for Data Processing and Analysis of the International Physical Activity Questionnaire (IPAQ) [Internet]. 2005. Available from: https://sites.google.com/site/theipaq/scoring-protocol

[34] Fransson E, De Faire U, Ahlbom A, Reuterwall C, Hallqvist J, Alfredsson L (2004). The risk of acute myocardial infarction: interactions of types of physical activity. Epidemiology.

[35] Lee IM, Paffenbarger RS (2000). Associations of light, moderate, and vigorous intensity physical activity with longevity: the Harvard alumni health study. Am J Epidemiol.

[36] Department of Human and Health Services. Physical Activity Guidelines for Americans. 2nd ed. Washington: Health.gov; 2018.

[37] Elosua R, Redondo A, Segura A, Fiol M, Aldasoro E, Vega G (2013). Dose-response association of physical activity with acute myocardial infarction: do amount and intensity matter?. Prev Med (Baltim).

[38] Kwak L, Hagströmer M, Sjostrom M (2012). Can the IPAQ-long be used to assess occupational physical activity?. J Phys Act Health.

